# Integrated mRNA-MicroRNA Profiling of Human NK Cell Differentiation Identifies MiR-583 as a Negative Regulator of IL2Rγ Expression

**DOI:** 10.1371/journal.pone.0108913

**Published:** 2014-10-14

**Authors:** Sohyun Yun, Su Ui Lee, Jung Min Kim, Hyun-Jun Lee, Hae Young Song, Young Kyeung Kim, Haiyoung Jung, Young-Jun Park, Suk Ran Yoon, Sei-Ryang Oh, Tae-Don Kim, Inpyo Choi

**Affiliations:** 1 Immunotherapy Research Center, Korea Research Institute of Bioscience and Biotechnology, Daejeon, Republic of Korea; 2 Natural Medicine Research Center, Korea Research Institute of Bioscience and Biotechnology, Ochang-eup, Republic of Korea; 3 NAR Center, Inc., Daejeon Oriental Hospital of Daejeon University, Daejeon, Republic of Korea; 4 Department of Functional Genomics, Korea University of Science and Technology, Daejeon, Republic of Korea; Duke University, United States of America

## Abstract

Natural killer (NK) cells are innate immune effector cells that protect against cancer and some viral infections. Until recently, most studies have investigated the molecular signatures of human or mouse NK cells to identify genes that are specifically expressed during NK cell development. However, the mechanism regulating NK cell development remains unclear. Here, we report a regulatory network of potential interactions during *in vitro* differentiation of human NK cells, identified using genome-wide mRNA and miRNA databases through hierarchical clustering analysis, gene ontology analysis and a miRNA target prediction program. The microRNA (miR)-583, which demonstrated the largest ratio change in mature NK cells, was highly correlated with IL2 receptor gamma (IL2Rγ) expression. The overexpression of miR-583 had an inhibitory effect on NK cell differentiation. In a reporter assay, the suppressive effect of miR-583 was ablated by mutating the putative miR-583 binding site of the IL2Rγ 3′ UTR. Therefore, we show that miR-583 acts as a negative regulator of NK cell differentiation by silencing IL2Rγ. Additionally, we provide a comprehensive database of genome-wide mRNA and miRNA expression during human NK cell differentiation, offering a better understanding of basic human NK cell biology for the application of human NK cells in immunotherapy.

## Introduction

Natural killer (NK) cells are lymphocytes that can eliminate cancer and some viral infections without prior sensitization by targeting major histocompatibility complex (MHC) antigens on target cells through their effector functions, such as cytotoxicity and cytokine secretion [1]. Human NK cells, granular CD56^+^CD3^−^ lymphocytes, are derived from CD34^+^ hematopoietic stem cells (HSCs) in the bone marrow (BM) and are subsequently differentiate into fully functional mature NK cells (mNK) in peripheral tissue microenvironments, such as the fetal thymus [1], [2]. During NK cell development process, these cells acquire optimal cytolytic and effector abilities depending on the balance between activating and inhibitory receptors. The determination of intermediates in the development of NK cells is primarily dependent on NK cell surface markers, including CD56 and killer inhibitory receptors (KIRs) in humans and NK1.1, DX5, and Ly49 in mice [1]. Although developmental intermediates in human T and B cells have been reasonably well defined, our knowledge about the *in vivo* stages of human NK cell development is very limited [3]. Recently, Aharon G. Freud *et al.* suggested that NK cells differentiate through four discrete intermediate stages in secondary lymphoid tissue: stage 1, CD34^+^CD117^−^CD94^−^, stage 2, CD34^+^CD117^+^CD94^−^, stage 3, CD34^−^CD117^+^CD94^−^, and stage4, CD34^−^CD117^+/−^CD94^+^ [4].

Most studies have identified genes that are closely related to NK cell development and function using mouse knockout (KO) models of the transcription factors (TFs) that modulate cell surface marker expression during NK cell differentiation. The TFs Ikaros [5], Ets-1 [6], PU.1 [7] and Id2 [8] are essential for the proliferation and differentiation of mature NK cells. Additionally, TFs such as GATA-3 [9], T-bet [10] and IRF-2 [11] appear to be involved in NK cell maturation. Furthermore, since the advent of *in vitro* protocols that analyze cytokine-mediated NK differentiation from HSCs, recent studies have demonstrated that important genes such as TOX [12] and IGF-1 [13] regulate human NK cell development. In these processes, interleukin-15 (IL-15) is an essential cytokine that stimulates the development and expansion of NK cells in humans and mice. Interestingly, IL-15 KO mice failed to develop functional, mature NK cells [14]. In addition, mice with impaired STAT5 or Jak3, which can modulate IL-15 signaling, showed defects in NK cell development [14].

MicroRNAs (miRNAs) are endogenous short non-coding RNAs (19–22 nt) that inhibit the expression of target genes by binding to the 3′ UTR of specific target mRNAs in eukaryotic cells. Recently, the involvement of miRNAs in immune responses and the development of immune cells from HSCs have been widely investigated manipulating specific miRNAs levels [15], [16] or disrupting molecules involved in the biogenesis/activity of all miRNAs, such as Argonaute [17], Drosha [18] and Dicer [19]–[22]. These genetic studies have demonstrated that miRNAs play essential roles in immune cell development and function [15], [23], [24]. In a previous study, miR-150 was reported to regulate the development of NK cell using miR-150 KO mice [25]. MiR-155 transgenic (tg) mice had increased numbers of NK cell and enhanced survival of NK cells; however, miR-155-deficient mice showed defects in NK cell maintenance and maturation at steady state [26], [27].

In humans, miR-483-3p has been validated as a negative regulator of human NK cell development and cytotoxicity by targeting IGF-1 [13]. Moreover, miR-27a* has been shown to negatively regulate NK cell cytotoxicity by silencing the expression of Prf1 and GzmB, which are essential effector molecules for human NK cell cytotoxicity [28]. Despite evidence for a broad role in regulating immune function, the molecular mechanisms regulated by miRNAs during the development of human NK cells remains poorly understood [24], [29], [30].

Here, we performed genome-wide mRNA and miRNA arrays and analyzed the resulting data through hierarchical clustering analysis, gene ontology analysis and miRNA target prediction programs. Our data show the highly correlated target mRNAs for predicted miRNAs and identify miR-583 as a negative regulator of NK cell differentiation through its ability to silence IL2Rγ.

## Materials and Methods

### Cell preparation and culture

Umbilical cord blood (UCB) samples were provided by Chungnam National University Hospital (Daejeon, Republic of Korea). Samples of human CB were obtained from umbilical veins of normal and full-term infants after written informed consent by their mothers, and the protocol was approved by the guidance of the Korea Research Institute of Bioscience and Biotechnology (KRIBB) Institutional Review Board (KRIBB-IRB-20051216-05). *In vitro* NK cell differentiation from CD34^+^Lin^−^ was performed as previously described [31]. Briefly, the isolated CD34^+^Lin^−^ were maintained in MyeloCult H5100 supplemented with stem cell factor (SCF, 30 ng/ml) and flt-3 ligand (FL, 50 ng/ml) for 14 days at 37°C, 5% CO2. The culture medium was refreshed every 3 days. Then, the medium was changed to differentiation medium containing human IL-15 (30 ng/ml, R&D Systems) and cultured for another 14 days. Every 3–4 days, half of the medium was discarded and replenished by fresh medium containing freshly added cytokines. MyeloCult H5100 (Stem Cell Technologies) supplemented with 10^−6^ M freshly dissolved hydrocortisone (HC, Sigma) and 50 µg/ml gentamicin was used as culture medium.

### RNA isolation

Total RNA from each sample was extracted using TRIZOL reagent (GibcoBRL, Rockville, MD, USA) according to the protocol of the manufacturer. RNA was treated with the RNase-free DNase I (Promega, Madison, WI, USA) to reduce DNA contamination. Total RNA concentration and purity were determined spectrophotometrically by the absorbance ratio at 260∶280 nm 1.8 or more. The integrity of RNA samples was also confirmed by appearance of distinct 28S and 18S bands of ribosomal RNA using Bioanalyzer 2100 system (Agilent Technology, Santa Clara, CA, USA).

### Flow cytometry and Ab used

Cell sorting was performed on the FACSAria (BD Bioscience), and phenotypic analysis was performed on the FACS Canto II (BD Bioscience) using CellQuest Pro Software (BD bioscience and FlowJo software. For cell-surface staining, collected cells were washed twice with ice-cold PBS followed by incubation with saturating concentrations of the appropriate mAbs for 15 min at 4°C and then, were washed twice in ice-cold PBS. For intracellular staining, cells were fixed and rendered permeable using the Fix and Perm kit (BD biosciences), according to the manufacturer's instructions. The antibodies used in this study were FITC-conjugated CD34, CD3 and NKp46, PE-conjugated CD56, NKG2D, NKp30, CD3, CD117, CD132, CD107a and p-STAT5, APC-conjugated CD56, CD94, and APC-Cy7 conjugated CD56 (BD bioscience).

### Gene expression microarray

For control and test RNAs, the synthesis of target cRNAs and hybridization were performed using Agilent's Low RNA Input Linear Amplification Kit PLUS (Agilent Technology) according to the manufacturer's instructions. Briefly, each 0.5 µg total RNA was mixed with the diluted Spike mix and T7 promoter primer mix and incubated at 65°C for 10 min. cDNA master mix (5× First strand buffer, 0.1 M DTT, 10 mM dNTP mix, RNase-Out, and MMLV-RT) was prepared and added to the reaction mixer. The samples were incubated at 40°C for 2 h and then the RT and dsDNA synthesis was terminated by incubating at 65°C for 15 min. The transcription master mix was prepared as the manufacturer's protocol (4× Transcription buffer, 0.1 M DTT, NTP mix, 50% PEG, RNase-Out, inorganic pyrophosphatase, T7-RNA polymerase, and Cyanine 3/5-CTP). Transcription of dsDNA was performed by adding the transcription master mix to the dsDNA reaction samples and incubating at 40°C for 2 h. Amplified and labeled cRNA was purified on RNase Mini Spin Columns (Qiagen, Hilden, Germany) according to the manufacturer's protocol. Labeled cRNA target was quantified using ND-1000 spectrophotometer (NanoDrop Technologies, Wilmington, DE, USA). After checking labeling efficiency, each 750 ng of cyanine 3-labeled and cyanine 5-labeled cRNA target were mixed and the fragmentation of cRNA was performed by adding 10X blocking agent and 25X fragmentation buffer and incubating at 60°C for 30 min. The fragmented cRNA was resuspended with 2X hybridization buffer and directly pipetted onto assembled Agilent Whole Human Genome Oligo Microarray (44K). The arrays hybridized at 65°C for 17 h with 10 rpm rotating in Agilent Hybridization Oven. The hybridized microarrays were washed as the manufacturer's washing protocol (Agilent Technology). The mRNA microarray data have been submitted to the Gene Expression Omnibus [5] database (GEO accession numbers: GSE47521).

### MiRNAs expression microarray

For control and test RNAs, the labeling of target miRNAs and hybridization were performed using Agilent's miRNAs Labeling Reagent and Hybridization Kit (Agilent Technology) according to the manufacturer's instructions. Briefly, each 100 ng of total RNA were dephosphorylated with 15 units of calf intestine alkaline phosphatase (CIP), followed by RNA denaturation with 40% DMSO and 10 min incubation at 100°C. Dephosphorylated RNA were ligated with pCp-Cy3 mononucleotide and purified with Micro Bio-Spin 6 Columns (Bio-Rad, Hercules, CA, USA). After purification, labeled samples were resuspended with Gene Expression Blocking Reagent and Hi-RPM Hybridization buffer, followed by boiling for 5 min at 100°C and 5 min chilled on ice. Finally, denatured labeled probes were pipetted onto assembled Agilent's Human miRNAs Microarray (15K) and hybridized at 55°C for 20 h with 20 rpm rotating in Agilent Hybridization Oven. The hybridized microarrays were washed as the manufacturer's washing protocol (Agilent Technology). The miRNAs microarray data have deposited as an Excel file in Table S3.

### Data acquisition and analysis

The hybridization images were analyzed by Agilent DNA microarray Scanner and the data quantification was performed using Agilent Feature Extraction software. The average fluorescence intensity for each spot was calculated and local background was subtracted. All data normalization and selection of fold-changed genes were performed using GeneSpring GX 7.3 (Agilent Technology). For gene expression microarray data, genes were filtered with removing flag-out genes in each experiment. In the gene expression microarray, intensity-dependent normalization (LOWESS) was performed, where the ratio was reduced to the residual of the Lowess fit of the intensity vs. ratio curve. The averages of normalized ratios were calculated by dividing the average of normalized signal channel intensity by the average of normalized control channel intensity. Functional annotation of genes was performed according to Gene Ontology Consortium (http://www.geneontology.org/index.shtml) by GeneSpring GX 7.3. Gene classification was based on searches done by GeneCards (http://www.genecards.org/), miRanda (http://www.microrna.org/), DAVID (http://david. abcc.ncifcrf.gov/), and Medline databases (http://www.ncbi.nlm.nih.gov/).

### MiRNA transfection

MiRNA mimic control, and miRNA mimics were purchased from Dharmacon RNA Technologies. Transfections of differentiating NK cell with miRNA mimic control, and miRNA mimics were performed by nucleofection using an Amaxa Human CD34 Cell Nucleofector kit (Lonza). In brief, 100 µL of one nucleofection sample contained 3×10^6^ cells and 100 nM (final concentration) miRNA mimic control, and miRNA mimics. These cells were subjected to nucleofection using program U-08 according to the manufacturer's instructions (Amaxa).

### NK cell functional assays

Cytotoxicity was examined using a standard 4-h ^51^Cr-release assay. ^51^Cr-labeled target cells (1×10^4^ cells/well) and serial dilution cells were used in triplicates. Radioactivity of the supernatant containing ^51^Cr was measured using a γ-counter. The percentage of specific lysis was calculated using the formula: (experimental release-spontaneous release)/(maximum release-spontaneous release) ×100. To evaluate cytokine secretion, differentiating NK cell (1×10^5^ cells/well) were stimulated in duplicate for 16 h with IL-18 (30 ng/ml), or PMA (phorbol 12-myristate 13-acetate)/IO (ionomycin) (1 or 2 ng/ml, 0.1 or 0.2 µg/ml). The secretion of IFN-γ (eBioscience), into the supernatant was measured by ELISA.

### Quantitative RT-PCR

For miR quantitative RT-PCR (qRT-PCR), cDNA was synthesized with the TaqMan MicroRNA Reverse Transcription Kit (Applied Biosystems, Carlsbad, CA), and primer/probe sets for miR-143, miR-223, miR-583, and miR-150, were purchased from Applied Biosystems. For quantitative real-time polymerase chain reaction (RT-PCR), total RNA was extracted using TRIzol (Invitrogen) and reverse transcribed into cDNA using M-MLV reverse transcriptase (Promega) with random primers (Takara Bio). Real-time PCR was performed using a Dice TP 800 Thermal Cycler and the SYBR Premix Ex Taq (Takara Bio). The data were normalized to the amount of glyceraldehyde 3-phosphate dehydrogenase (GAPDH) transcript. The primer sequences were as follows: 5′-cagcctcaagatcatcagca-3′ and 5′-gtcttctgggtggcagtgat-3′ for IL2Rγ, 5′-cagcctcaagatcatcagca-3′ and 5′-gtcttctgggtggcagtgat-3′ for GAPDH, 5′-cgtgaggtccgttaggaaaa-3′ and 5′-atagtgggatgcgagtccag-3′ for ID2, 5′-aaccaatcctgcttctgc-3′ and 5′-actgtcgtaataatggcgta-3′ for Granzyme B, 5′- catctgcctcttgggacct-3′ and 5′-agctctggacacagggtgag-3′ for AANAT, 5′- atgactgggcacaacagaca-3′ and 5′-agtgacaacgtcgagcacag-3′ for neomycin gene (Neo). 5′- gcagcagactctcccaaaac-3′ and 5′-tggcaacagatggtcacttg-3′ for NKp46, 5′-aggaggtgaggaatggaacc-3′ and 5′-tccactctgcacacgtagatg-3′ for NKp30, and 5′-tcggtcaagggaatttgaac-3′ and 5′-ttttcaacacgatggcaaaa-3′ for NKG2D.

### Immunoblot analysis

Cells were washed twice with ice-cold PBS and lysed in RIPA (50 mM Tris–HCl, pH 7.4, 150 mM NaCl, 0.25% SDS, 1% NP-40, and 1 mM EDTA, supplemented with a protease inhibitor cocktail tablet from Roche). The cell lysates were resolved on 8 or 12% SDS PAGE gels and transferred to PVDF membranes (Millipore). The membranes were probed with antibodies specific for AANAT (Cell Signaling) and GAPDH (Assay Designs). After incubation with peroxide-conjugated anti-rabbit IgG (Jackson Immuno-Research), signals were detected using SuperSignal West Pico Chemiluminescent Substrate.

## Results

### Differential expression profiling of mRNAs during *in vitro* differentiation of human NK cells

**Figure 1:**
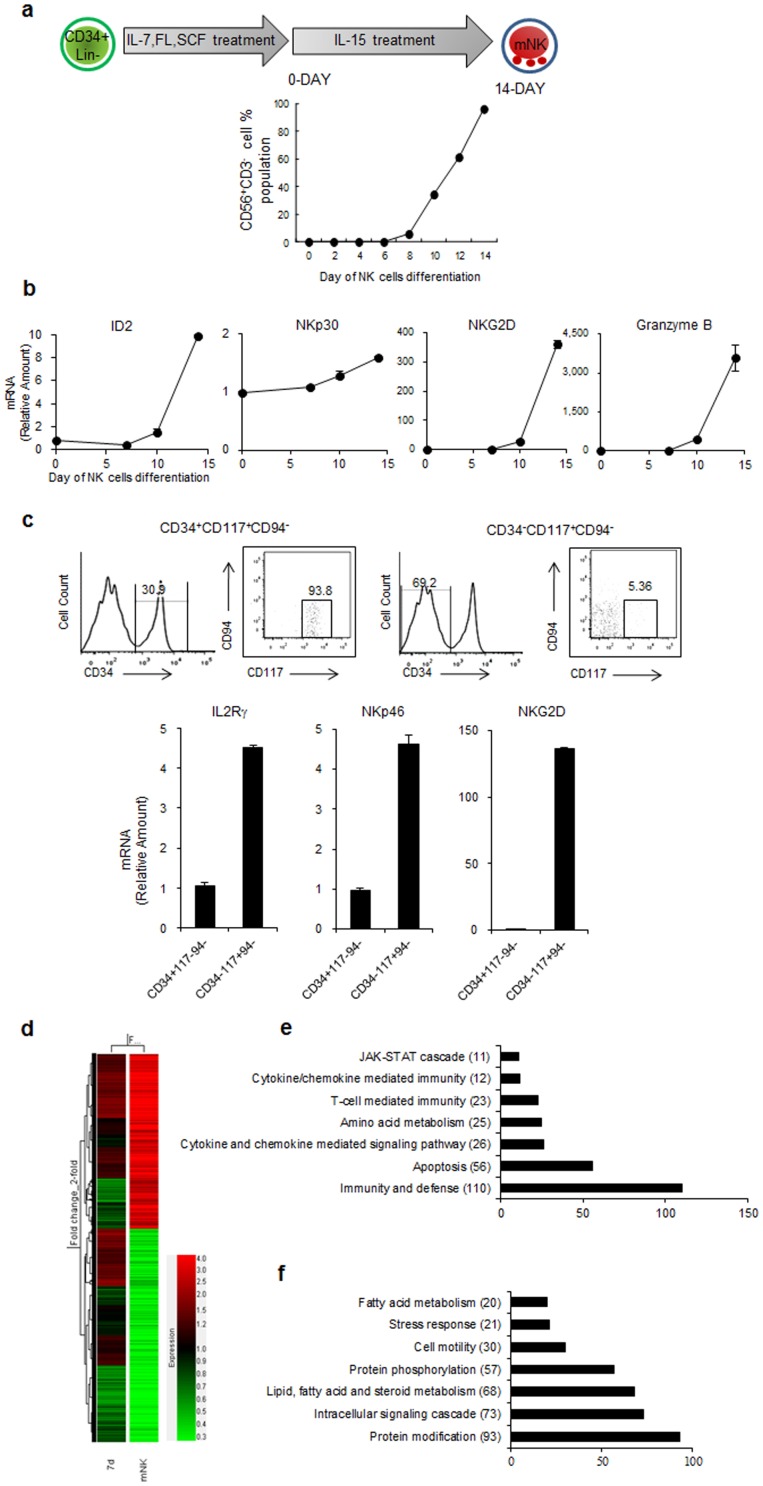
Genome-wide mRNA expression. (a) Isolated HPC (CD34^+^Lin^−^) cells were cultured in IL-15-supplemented media, and the culture media was replaced every 48 h. The expression of CD56 as an NK cell surface marker was analyzed using FACS. (b) The kinetics of the mRNA expression of Id2, NKp30, NKG2D and Granzyme B were analyzed by real-time qPCR. (c) Stage 2 progenitors (CD34^+^CD117^+^CD94^−^) and stage 3 progenitors (CD34^−^CD117^+^CD94^−^) were isolated from UCB by flow sorting. The mRNA expression of IL2Rγ, NKp46 and NKG2D were analyzed by real-time qPCR. (d) A dendrogram of hierarchical clustering revealed genes that were altered more than 2-fold in 7d- and 14-d (mNK) cultured NK cell compared with 1 d-cultured cells. (E-F) The bar graphs represent the top seven functional categories of upregulated (e) or downregulated (f) genes according to the gene ontology analysis (as determined by DAVID, as described in the Methods). The data are representative of three independent experiments performed using three different UCB samples and represent the mean values ± S.E.M. of duplicates.

Thus far, IL-15 has been known as an important cytokine for the differentiation of NK cells from HSCs *in vitro* [31]. We noted from previous work that the NK cell marker CD56 was detectable approximately 7 days (7d) after IL-15 supplementation during NK cell differentiation *in vitro* (Fig. 1a) [31]. To identify differentially expressed genes during human NK cell development, we performed mRNA arrays using total RNA isolated from 1-, 7- or 14-d (mNK) cultured cells that had been grown in media supplemented with IL-15 to induce their differentiation into NK cells. Next, we analyzed the expression kinetics of previously known NK cell markers that are induced during NK differentiation using real-time qPCR (Fig. 1b). The expression patterns of the NK cell related genes, Id2, NKp30, NKG2D, and GzmB were consistent with the CD 56 expression patterns; thus the time points used in our study could be considered suitable to explore the expression patterns of NK cell markers during NK cell development [1]. Additionally, we compared freshly isolated stage 2 progenitors (CD34^+^CD117^+^CD94^−^) and stage 3 progenitors (CD34^−^CD117^+^CD94^−^) from UCB for their relative mRNA expression of genes important for NK cell differentiation and activity. Consistent with the mRNA array data (Table 1 and Fig. 1b), we observed that the expressions of NKp46, NKG2D and IL2Rγ were up-regulated throughout the NK cell developmental stages *in vivo* (Fig. 1c). As shown in Fig. 1d, the expressions of 2920 total genes was altered more than 2-fold in the 7- and 14-d (mNK) cultured NK cells compared with the 1-d cultured cells using hierarchical clustering, on the other hand, 1335 genes were upregulated and 1585 genes were downregulated. All the microarray data are available in the Gene Expression Omnibus under accession GSE47521.

**Table 1:** The signaling genes related to immune system changed between 7 d- and 14d-cultured (mNK) cells.

Category	Gene symbol	Gene Name	24 h vs. 7 d	24 h vs. mNK	ratio	Genbank Acc. No.
**Receptor**	LAIR2	leukocyte-associated immunoglobulin-like receptor 2	1.61	45.94	28.6	NM_002288
	NCR3	natural cytotoxicity triggering receptor 3	1.43	20.4	14.3	NM_147130
	MARCO	macrophage receptor with collagenous structure	0.6	7.79	12.9	NM_006770
	ITGB7	integrin, beta 7	1.07	10.56	9.9	NM_000889
	SLAMF6	SLAM family member 6	1.16	9.92	8.6	NM_052931
	IL2RA	interleukin 2 receptor, alpha	1.12	9.35	8.3	NM_000417
	CD69	CD69 molecule	1.02	8.04	7.9	NM_001781
	IFNAR2	interferon (alpha, beta and omega) receptor 2	1.39	9.75	7	NM_207585
	CR1	complement component (3b/4b) receptor 1	1.26	6.79	5.4	NM_000651
	CLECL1	C-type lectin-like 1	0.58	3.05	5.3	NM_172004
	**EDNRB**	**endothelin receptor type B**	**1.35**	**0.07**	**20**	**NM_003991**
	**SPSB1**	**splA/ryanodine receptor domain**	**1.84**	**0.25**	**7.3**	**NM_025106**
	**CXCR2**	**chemokine (C-X-C motif) receptor 2**	**1.16**	**0.21**	**5.6**	**NM_001557**
**Cytokines**	C1QTNF6	C1q and tumor necrosis factor related protein 6	0.59	4.38	7.4	NM_031910
	TNF	tumor necrosis factor (TNF superfamily, member 2)	0.54	3.49	6.5	NM_000594
	CCL1	chemokine (C-C motif) ligand 1	1.84	11.57	6.3	NM_002981
	IFNG	interferon, gamma	1.6	9.46	5.9	NM_000619
	IL24	interleukin 24	1.52	8.97	5.9	NM_006850
	CCL22	chemokine (C-C motif) ligand 22	0.57	2.97	5.2	NM_002990
**Adaptor protein**	TIRAP	toll-interleukin 1 receptor (TIR) domain	1.34	16.53	12.3	NM_001039661
	LGALS3BP	lectin, galactoside-binding, soluble, 3 binding protein	0.95	11.39	12	NM_005567
	EDA	ectodysplasin A	1.08	6.15	5.7	NM_001399
	**RALGPS2**	**Ral GEF with PH domain and SH3 binding motif 2**	**1.4**	**0.16**	**8.8**	**NM_152663**
	**RAB3C**	**RAB3C, member RAS oncogene family**	**1.2**	**0.16**	**7.6**	**NM_138453**
	**SH3PXD2A**	**SH3 and PX domains 2A**	**1.28**	**0.17**	**7.5**	**NM_014631**
**Kinases**	ZAP70	zeta-chain (TCR) associated protein kinase 70 kDa	1.18	10.54	8.9	NM_001079
	CSNK2A2	casein kinase 2, alpha prime polypeptide	1.09	6.09	5.6	NM_001896
	**DGKG**	**diacylglycerol kinase, gamma 90 kDa**	**1.68**	**0.16**	**10.8**	**NM_001346**
	**PLK2**	**polo-like kinase 2 (Drosophila)**	**1.42**	**0.24**	**5.8**	**NM_006622**
	**SGK2**	**serum/glucocorticoid regulated kinase 2**	**0.64**	**0.12**	**5.1**	**NM_170693**
**Phosphatase**	**PPM1A**	**protein phosphatase, Mg2+/Mn2+ dependent, 1A**	**0.95**	**0.18**	**5.2**	**NM_177951**
**TF**	ETS1	v-ets erythroblastosis virus E26 oncogene homolog 1	0.51	4.7	9.2	NM_005238
	**STAT2**	**signal transducer and activator of transcription 2**	**0.53**	**0.05**	**10.9**	**NM_005419**

Next, to define the functional properties of those genes that were altered more than 2-fold according to the mRNA array data, the upregulated (Fig. 1e) and downregulated (Fig. 1f) genes were categorized using gene ontology classifications. Following these analyses, the major categories of upregulated genes were classified as “immunity and defense” genes, whereas the downregulated genes were identified as “protein modification” and “intracellular and signaling cascade” genes. To better understand the expression of signaling molecules during human NK cell development, we selected the genes that were categorized as “immunity and defense” and “intracellular and signaling cascade” genes and listed them in Table 1 according to their functional properties. As shown in Table 1, the list of 33 genes, which includes 22 upregulated genes and 11 downregulated genes (in bold), that had intensity ratio changes in excess of 5-fold contained receptors (NCR1 and NCR3), cytokines (IFN-γ and CCL1) and TFs (Ets-1) known to be involved in NK cell development and activity.

### Differential miRNA expression profiling during *in vitro* differentiation of human NK cells

**Figure 2:**
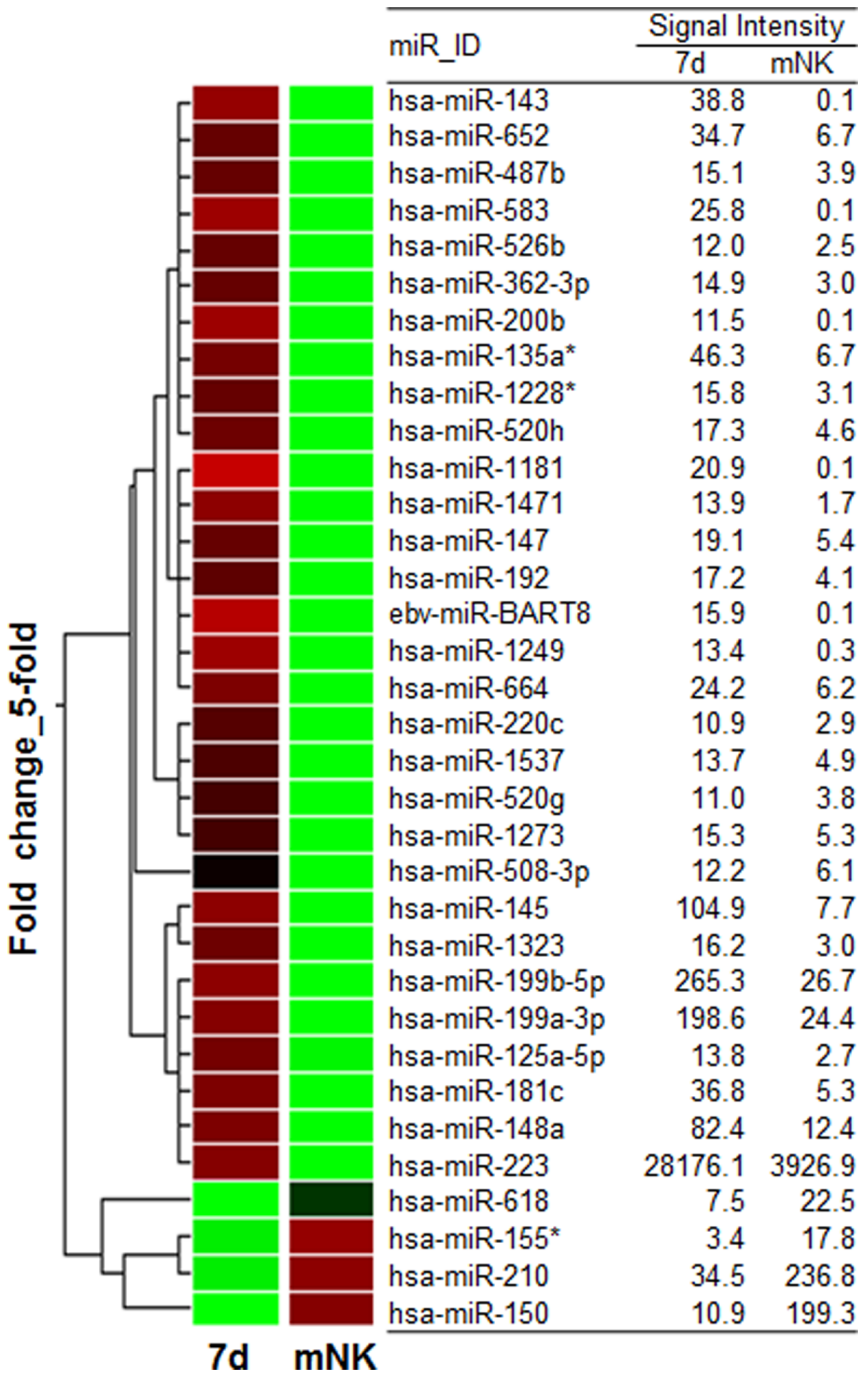
Genome-wide miRNAs expression profiles. A dendrogram showing hierarchical clustering for the miRNAs altered more than 5-fold in 7d-cultured cells compared with 14d-cultured (mNK) cells. The results are shown as the mean values of duplicate experiments.

Next, to investigate the relationship between miRNAs and their target mRNAs during NK cell development, we performed miRNA arrays using total RNA isolated from 7- or 14-d (mNK) cultured cells and deposited the summarized data in Table S3. As shown in Fig. 2, the expression of 34 miRNAs was found to be altered more than 5-fold in 14-d (mNK) cultured cells compared with the 7-d cultured cells using hierarchical clustering analysis. Of these 34 miRNAs, 4 miRNAs were upregulated and 30 were downregulated. In these results, we confirmed that the expression of miR-150 and miR-155* were strongly increased in mNK cells. Importantly, miR-150 was previously identified as a regulator of mouse NK cell development and cytotoxicity [25], [32], and miR-155 was critically required for NK cell maturation and maintenance at steady state [27]. Additionally, the downregulation of miR-223 in human mNK cells was previously reported to regulate GzmB translation during murine NK cell activation [33]. Thus, we hypothesized that individual miRNAs could be evaluated as biomarkers that regulate the expression of key molecular signatures during NK cell development.

Based on the data presented in Fig. 2, we chose the 4 miRNAs (miR-583, miR-143, miR-200b and miR-1181) that were significantly downregulated in mNK cell, which suggested an inhibition of target genes by the predicted miRNAs. As shown in Table 2, we have summarized the highly correlated target proteins for the 4 selected miRNAs using the miRNAs target prediction program miRanda. However, it should be noted that individual miRNAs interact with the conserved sites of multiple target genes and that most miRNAs and their potential target mRNAs do not necessarily match. To demonstrate the relationship between the four selected the miRNAs and their target mRNAs during NK cell differentiation, we next attempted to construct a regulatory network of potential interactions between the miRNAs and mRNAs identified during the expression analysis.

**Table 2:** The list of highly correlated genes between miRNA and mRNA expression between early (7 d) and mNK cells.

Gene Symbol	7d	mNK	Genbank Acc. No.	Predicted miRNA
NDFIP2	0.86	10.43	NM_019080	hsa-miR-583, has-miR-143
YTL3	0.88	7.01	NM_001009991	hsa-miR-583
MYBL1	0.74	6.36	NM_001080416	hsa-miR-200b,has-miR-143
SYTL3	0.70	6.35	NM_001009991	hsa-miR-583
AREG	0.67	5.51	NM_001657	hsa-miR-583
FUT8	0.92	4.92	NM_178155	hsa-miR-583
PPP4R2	0.98	4.76	NM_174907	hsa-miR-200b
KDELC1	0.82	4.72	NM_024089	hsa-miR-200b
NFATC2	0.36	4.66	AK025758	has-miR-143
RSAD2	0.85	4.38	NM_080657	hsa-miR-200b
LPIN1	0.69	3.89	NM_145693	hsa-miR-200b
PPP4R2	0.98	3.82	NM_174907	hsa-miR-200b
MLL5	0.62	3.78	NM_182931	hsa-miR-200b,has-miR-143
NSMCE1	0.85	3.66	NM_145080	hsa-miR-583
MYBL1	0.40	3.53	NM_001144755	hsa-miR-200b,has-miR-143
TTC19	0.66	3.15	NM_017775	hsa-miR-583,hsa-miR-200b
ELMO2	0.98	3.15	NM_182764	hsa-miR-200b
CACNG7	0.74	3.11	NM_031896	hsa-miR-583
XPNPEP1	0.82	3.01	NM_020383	hsa-miR-583
HDAC4	1.00	2.96	NM_006037	hsa-miR-200b,hsa-miR-1181
DIDO1	0.97	2.82	NM_022105	hsa-miR-200b
CAPRIN2	0.91	2.69	NM_001002259	hsa-miR-200b
FN1	0.96	2.66	NM_212482	hsa-miR-583
FYN	0.29	2.64	NM_002037	hsa-miR-583,hsa-miR-200b
CASK	0.98	2.64	NM_001126054	hsa-miR-583,hsa-miR-200b,has-miR-143
SHMT2	0.79	2.63	NM_005412	hsa-miR-583
CCDC50	0.61	2.60	NM_174908	hsa-miR-200b
C1orf97	0.97	2.60	NR_026761	hsa-miR-583,hsa-miR-200b
IL2RG	0.83	2.58	NM_000206	hsa-miR-583,has-miR-143
TBC1D22A	0.54	2.54	NM_014346	hsa-miR-583
XPNPEP1	0.83	2.49	NM_020383	hsa-miR-583
MSI2	0.94	2.43	NM_138962	hsa-miR-583has-miR-143
ASB4	0.89	2.41	NM_016116	hsa-miR-583,hsa-miR-200b
FAM127A	0.95	2.40	NM_001078171	hsa-miR-200b
SLC39A8	0.91	2.28	NM_022154	hsa-miR-200b
SLFNL1	0.72	2.27	NM_144990	has-miR-143
CTSC	0.99	2.26	NM_148170	hsa-miR-583,hsa-miR-200b
CLASP2	0.49	2.23	AJ288059	hsa-miR-200b
POLH	0.72	2.23	NM_006502	hsa-miR-583hsa-miR-200bhsa-miR-1181
CLDND1	0.90	2.22	NM_001040199	hsa-miR-583,hsa-miR-200b
SLC25A15	0.54	2.20	NM_014252	has-miR-143
TMEM14A	0.78	2.18	NM_014051	hsa-miR-200b
MCTP2	0.65	2.17	AL832717	hsa-miR-583,hsa-miR-200b,hsa-miR-1181
MYC	0.81	2.15	NM_002467	hsa-miR-200b
NEUROD2	0.97	2.09	NM_006160	hsa-miR-583,hsa-miR-1181
GPR18	0.73	2.01	NM_005292	hsa-miR-583,has-miR-143
YTHDC1	0.48	2.01	NM_001031732	hsa-miR-583,hsa-miR-200b

### A regulatory network of NK cell differentiation using the integrated analysis of miRNA-mRNA microarray data

**Figure 3:**
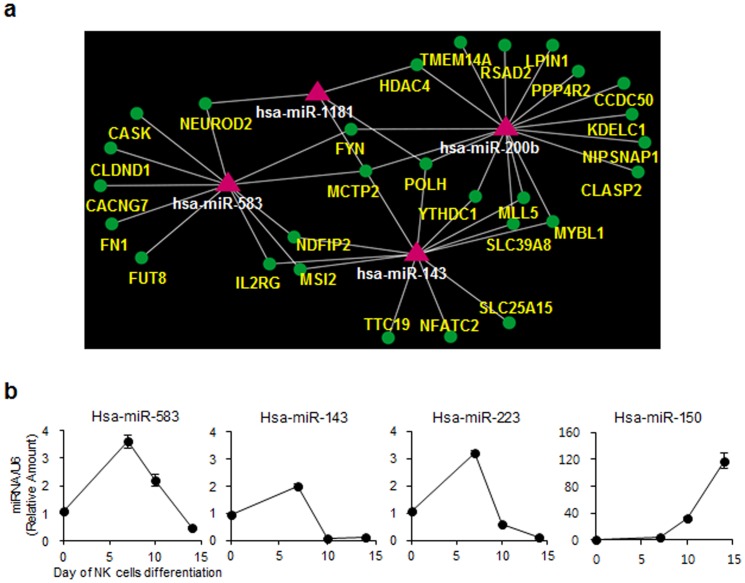
A regulatory network of NK cell differentiation derived from an integrated analysis of miRNAs-mRNA microarray data. (a) Negatively correlated miRNA-mRNA interactions were visualized as a network using Magia (miRNAs and genes integrated analysis web-based tool). This network provides for the first time a theoretical outline of the concerted action of regulating miRNAs (red triangles) and their potential target mRNAs (green circles). (b) Isolated HPC (CD34^+^Lin^−^) cells were cultured as described in the Materials and Methods. After being cultured in IL-15-supplemented media, the cells were collected at the indicated time intervals. The expression of miR-143, miR-223, miR-150 and miR-583 was analyzed by real-time qPCR. The data are representative of five independent experiments performed using two different UCB samples and represent the mean values ± S.E.M. of duplicates.

Recently, a study on the epigenetic regulation of gene expression reported that specific miRNA expression changes might contribute to distinct mRNA expression profiles, suggesting that miRNAs inhibit the expression of target genes via a negative relationship [34], [35]. Thus, we showed that negatively correlated miRNA-mRNA interactions could be visualized as a network; in this study, we used Magia (miRNA and genes integrated analysis web-based tool). As shown in Fig. 3a, this network gives information on the regulatory mechanisms between the largely suppressed four miRNAs (red triangles) in mNK cell and their potential target mRNAs (green circles). Importantly, both miR-583 and miR-143 were highly correlated with subunit IL2Rγ of the IL2 receptor, related to the IL-15 signaling pathway. In fact, IL2Rγ is essential for NK cell development, and it has been shown that IL2Rγ-deficient mice were completely devoid of NK cells [36].

Prior to validating whether miR-583 and miR-143 contributed to targeted suppression of IL2Rγ expression, we analyzed the expression kinetics of miR-583 and miR-143, as well as the well-known miRNAs miR-223 and miR-150, during NK cell differentiation using real-time qPCR (Fig. 3b). Our results were consistent with the microarray data presented in Fig. 2 showing that the expression of miR-583, miR-143 and miR-223 were decreased; however, the expression of miR-150 was increased during NK cell differentiation. Given these collective data, we hypothesized that miR-583 or miR-143 may play a role in NK cell differentiation through the regulation of IL2Rγ expression.

### Involvement of microRNA in IL2Rγ expression in human NK cells

**Figure 4:**
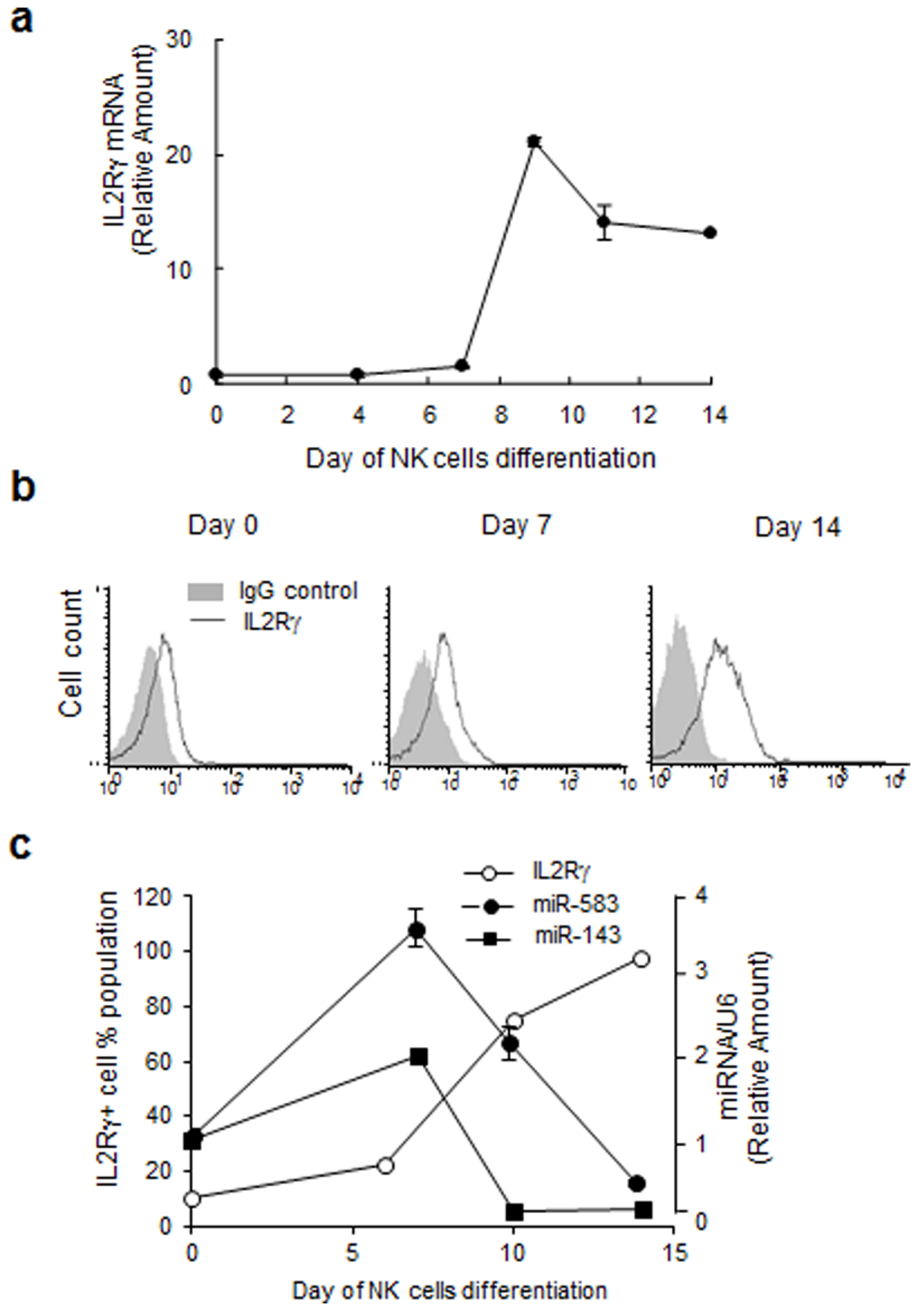
Possible involvement of microRNAs in IL2Rγ expression during human NK cell differentiation. Isolated CB HPCs (CD34^+^Lin^−^ cells) were cultured as described in the Materials and Methods. After being cultured in IL-15-supplemented media, the cells were collected at 48 h intervals. (a) The expression of the IL2Rγ gene was analyzed by real-time quantitative RT-PCR. (b) The expression of IL2Rγ protein was analyzed by FACS. (c) Expression profiling of IL2Rγ, miR-583 and miR-143 after IL-15 treatment. The data are representative of three independent experiments performed using five different UCB samples and represent the mean values ± S.E.M. of duplicates.

To determine whether IL2Rγ expression is regulated by the miR-583 and miR-143 miRNAs as predicted, we examined the mRNA and protein expression of IL2Rγ during NK cell differentiation. IL2Rγ mRNA expression was dramatically increased after 7d of IL-15 treatment, after which it was slightly downregulated during the final maturation into mNK cells (Fig. 4a and 4b). IL2Rγ mRNA transcript expression increased during NK cell differentiation and peaked at 9 d after IL-15 treatment. By contrast, miR-583 expression peaked at 7d after IL-15 treatment but was dramatically reduced during NK differentiation, implying that posttranscriptional regulation is involved in the expression of this receptor molecule (Fig. 4c). Although IL-15–related NK cell differentiation is closely associated with the expression of the IL2R complex, little is known about the mechanism regulating receptor expression on NK cells.

### Effects of miR-583 on differentiation and functional activity of NK cells

**Figure 5:**
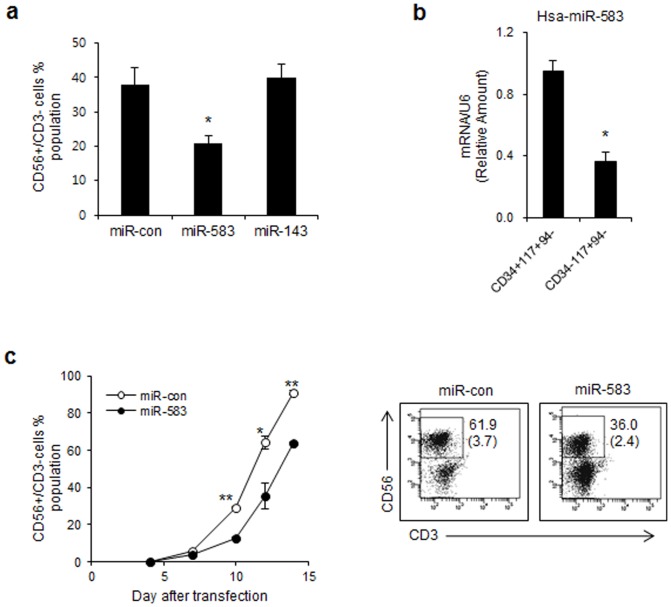
Human miR-583 downregulates NK cell differentiation. (a) Differentiating cells were transfected with miR-143, a miR-583 mimic or negative control miRNA. NK cell populations (CD56^+^CD3^−^ cells) were analyzed by FACS 10 days after transfection. *, *p*<0.1. (b) The expression level of miR-583 was decreased during NK cell development as shown by qRT-PCR. The results are shown as the mean expression values normalized against stage 2 (CD34^+^CD117^+^CD94^−^). *, *p*<0.1. (c) NK cell populations (CD56^+^CD3^−^ cells) were analyzed by FACS after miR-583 transfection at regular intervals during NK cell differentiation. The absolute numbers of differentiated NK cells are shown in the parentheses (×10^5^). *, *p*<0.01 **, *p*<0.001. The data are representative of three independent experiments performed using three different UCB samples and represent the mean values ± S.E.M. of duplicates.

To investigate the biological effects of miRNAs on NK cell development, miR-583 and miR-143 were validated as regulators of NK cell differentiation by targeting IL2Rγ. Although IL-15 receptor-mediated signaling is important for NK cell differentiation, it is not known whether miRNAs regulate IL-15 receptor expression during NK cell differentiation. To evaluate whether overexpression of miRNAs caused the selective reduction of IL2Rγ we transfected synthetic miRNA mimics into differentiating NK cells (0 day). We transfected synthetic miR-583 mimics into differentiating NK cells (0 day). Then, the medium was changed to differentiation medium containing human IL-15 (30 ng/ml). The introduction of miR-583 mimics led to an approximately 2-fold decrease in the percentage of mature CD56^+^CD3^−^ NK cell by the 10 d after transfection compared with the mimic control (Fig. 5a and 5c). In contrast, the introduction of a miR-143 mimic resulted in similar percentages of mature CD56^+^CD3^−^ NK cells and mimic controls (Fig. 5a). Thus, we focused on miR-583 as a regulator of NK cell differentiation *via* the IL-15 signaling pathway.

Next, we compared freshly isolated stage 2 progenitors (CD34^+^CD117^+^CD94^−^) and stage 3 progenitors (CD34^−^CD117^+^CD94^−^) from UCB for their relative miR-583 expression. Consistent with the miRNAs array data (Fig. 2), we observed that the miR-583 transcript level was decreased throughout the primary NK cell developmental stage *in vivo* (Fig. 5b).

**Figure 6:**
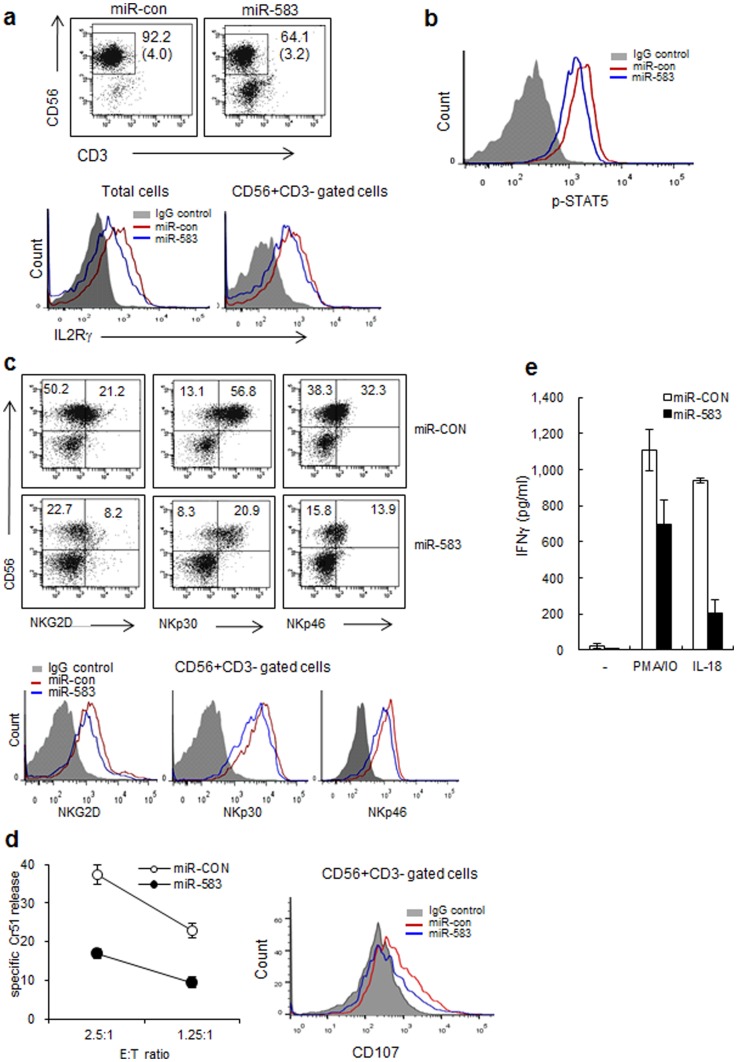
Human miR-583 downregulates NK cell activation by silencing IL2Rγ during NK cell differentiation. (a) The expression of IL2Rγ was analyzed by FACS 14 days after miR-control or miR-583 transfection in differentiating NK cells and CD56^+^CD3^−^ gated NK cells. Gray, IgG control; blue, miR-control; red, miR-583. The absolute numbers of differentiated NK cells are shown in the parentheses (×10^5^). (b) The expression of p-STAT5 was analyzed by FACS 7 days after miR-control or miR-583 transfection in differentiating NK cells. Gray, IgG control; blue, miR-control; red, miR-583. (c) The expression levels of NK cell activation receptors in differentiating NK cells and CD56^+^CD3^−^ gated NK cells were analyzed by FACS 14 days after miR-control or miR-583 transfection. Gray, IgG control; blue, miR-control; red, miR-583. (d). Cytotoxicity of differentiating NK cells was determined by ^51^Cr release assay against K562 cells at the indicated effector/target (E:T) ratios. *, *p*<0.1. The expression levels of CD107a in differentiating NK cells and CD56^+^CD3^−^ gated NK cells were analyzed by FACS. Gray, IgG control; blue, miR-control; red, miR-583. (e) Differentiating NK cells were stimulated with PMA/IO or IL-18. After 16 h, the supernatants were assayed for IFN-γ production by ELISA. *, *p*<0.1. Values represent the mean % of positive cells ± S.E.M. of triplicates. The data are representative of three independent experiments performed using three different UCB samples and represent the mean values ± S.E.M. of duplicates.

To determine whether the expression of IL2Rγ protein was suppressed by miR-583, we investigated the expression level of IL2Rγ on transfected differentiating NK cell by FACS. Differentiating NK cells transfected with miR-583 mimics showed decreases in IL2Rγ protein levels in both total cells and CD56^+^CD3^−^ gated cells (Fig. 6a). The signal transduction initiated by IL-15 involves tyrosine phosphorylation of STAT5, an essential transcription factor that mediates IL2R, by JAK3 in NK cells [37]. Therefore, we analyzed the expression level of p-STAT5 in differentiating NK cells by FACS 7 days after miR-control or miR-583 transfection. As shown in Fig. 6b, differentiating NK cell transfected with miR-583 mimics showed decreased levels of STAT5 phospholylation, compared with the differentiating NK cells transfected with the miR-control. We next considered the possibility that differentiating NK cell transfected with miR-583 mimics may have decreased expression of IL-15 dependent activation receptors because of reduced expression of p-STAT5 [38]. The expression levels of NK cell activation receptors decreased in differentiating NK cells transfected with miR-583 and in CD56^+^CD3^−^ gated NK cells (Fig. 6c).

Next, to confirm whether the differentiating NK cells transfected with miR-583 had full functional activity, we examined their functions such as cytolytic activity or production of cytokines. The miR-583-treated differentiating NK cells showed a markedly decreased capacity to kill K562 and produce IFN-γ. The reduced functional activities in miR-583 treated differentiating NK cells may reflect a relatively small population of NK cells by treatment of miR-583 compared with controls (Fig. 6d and 6e). Lysosomal-associated membrane protein-1 (LAMP-1 or CD107a) lines the membrane of cytolytic granules and is used as a marker of NK cell degranulation [39]. The expression of activating receptors is also crucial for NK-mediated killing of various target cells [40]. Thus, to investigate single cell–based assay for NK cytolytic activity, we analyzed the expression level of CD107a, and activating receptors in differentiating NK cells transfected with miR-583 or control miR. The CD56^+^CD3^−^ gated NK cell population showed the expression levels of CD107a and activating receptors, including NKG2D, NKp30 and NKp46, were decreased in miR-583-overexpressed NK cells compared with those of the miR-control (Fig. 6c and 6d). Taken together, these results suggest that the decreased expression of miR-583 plays an important role in the differentiation and function of human NK cell through the de-repression of IL2Rγ expression.

### Human miR-583 serves as a regulator of IL2Rγ protein expression in differentiating NK cells

**Figure 7:**
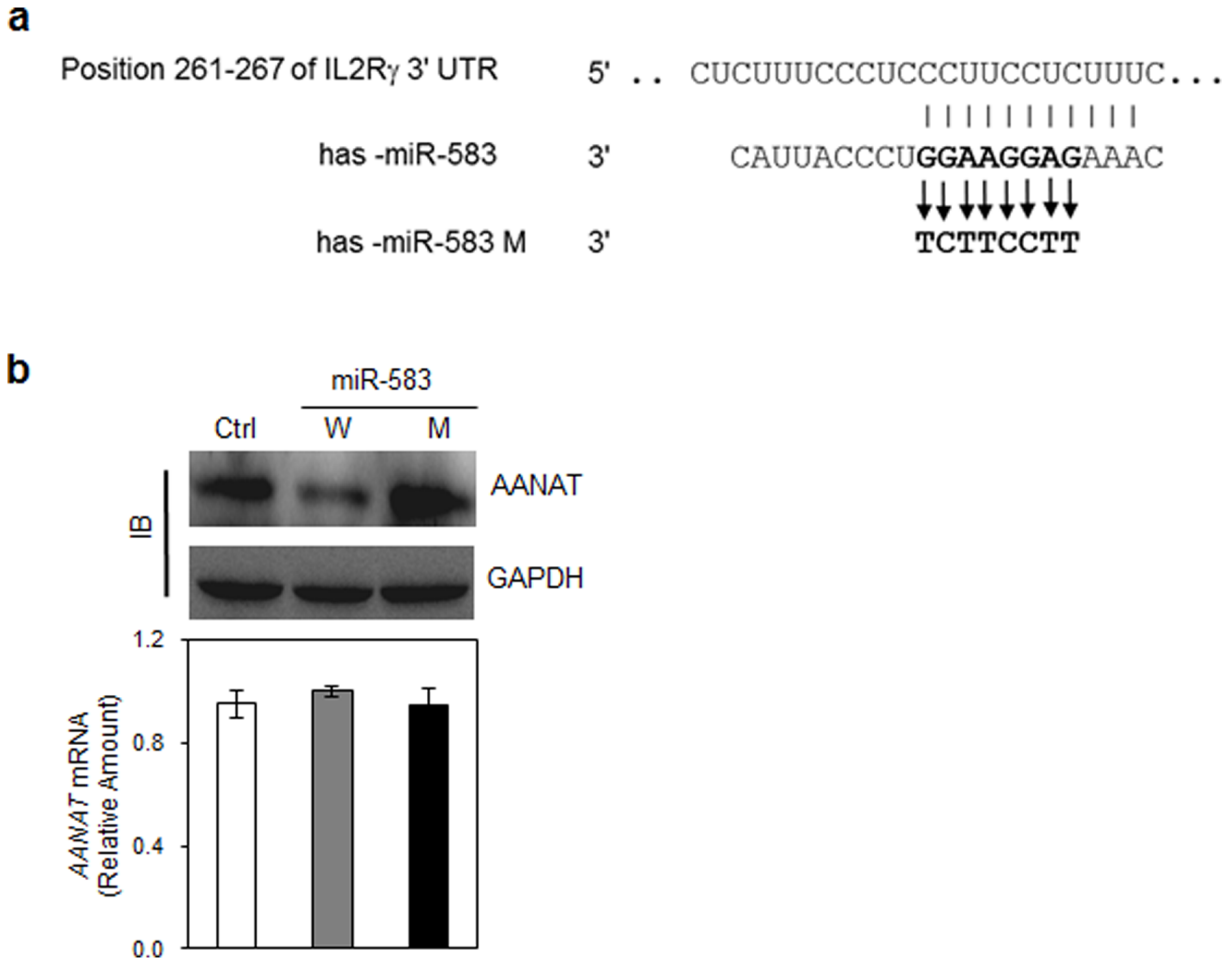
Human miR-583 specifically targets IL2Rγ 3′ UTR sequences. (a) Predicted miR-583 binding sites. Mutants (M). Point mutations are in bold. Numbers indicate the positions of nucleotides in the 3′ UTR. (b) Reporter assay using immunoblotting (IB) analysis. HEK-293FT cells were cotransfected with combinations of reporter plasmids containing IL2Rγ 3′ UTRs and miR-583 or mutant miR-583 (miR-583 M). Reporter AANAT mRNA levels were normalized to Neo mRNA levels as an internal control for the vector. The net amount of translated AANAT protein was determined by IB. GAPDH served as a loading control. The data are representative of two independent experiments (mean values ± S.E.M. of triplicates).

To further test whether miR-583 specifically targets the IL2Rγ, we performed reporter assays in cell cultures as described previously [28]. Although the overexpression of miR-583 in HEK-293FT cells dramatically reduced the expression of an AANAT reporter gene construct containing the wild-type IL2Rγ 3′ UTR (Fig. 7a and 7b), the ectopic expression of a control miRNA (Ctrl_miR) had no significant effect on the expression of these reporters (Fig. 7b). Moreover, point mutations in miR-583 induced the recovery of reporter gene expression without changing the AANAT mRNA levels (Fig. 7b). For these experiments, the reporter AANAT mRNA levels were normalized to neomycin resistance (Neo) gene mRNA as an internal control and showed little difference among the samples. These data suggest that miR-583 downregulates IL2Rγ expression by specifically targeting its 3′ UTR sequences.

## Discussion

In this study, we demonstrated a regulatory network of potential interactions between miRNA and mRNA expression during NK cell development induced by IL-15 treatment. First, we summarized the gene ontology classifications for the mRNA microarray expression profiles during the development of human NK cell. In these studies, we identified well-known marker genes, including cytokines, receptors and TFs (Table 2 and Table S1). Additionally, we found several key components of the cytotoxic NK cell machinery, including Prf1, GzmA, GzmB and CTSW. The KIR or KLR family members, IL2 receptor subunits (IL2Rα, IL2Rβ and IL2Rγ), NCR1 and CXCR3 are known as activating receptors that send activating signals to NK cells. The chemokines XCL1 and CCL3 (also known as MIP-1α) induce immune responses against pathogen infection.

In previous study, developmental process of mouse NK cells appears to be the transcriptional changes of genes associated with proliferation and effector function according to surface density of CD11b/CD27 [41]. As shown in Table S1, the expression of cyclin-dependent kinase (CDK) 14 that regulates cell cycle progression and cell proliferation [42] was decreased in mature NK cells whereas expressions of effector proteins such as GzmB and Prf1 were increased according to increment of CD56 expression. Furthermore, the expression of dual-specificity phosphatase (DUSP) family, controlling MAPKs, which is associated with cellular proliferation and differentiation increased in mature NK cells [43]. In these results, we suggest that maturation of NK cell in both mouse and human is accompanied by transcriptional changes of genes related to cell proliferation and acquisition of effector function.

For greater insight into the data, we performed signal pathway analysis using the KEGG pathway mapping tool (Table S2) based on the gene ontology classifications presented in Fig. 1d. These results suggested that 64 total genes (28 upregulated and 27 downregulated genes) are related to the PI3K-Akt signaling pathway, which regulates the balance between survival and apoptosis. Additionally, we showed that 138 total genes (19 upregulated and 3 downregulated genes) related to NK cell-mediated cytotoxicity pathways were altered. Thus, these data suggest that there were important changes in the expression of genes involved in cell proliferation and differentiation rather than genes involved in NK cell activation during NK cell development. Genome-wide mRNA array data examining key molecular signatures during human NK cell differentiation could provide additional important information.

Interestingly, human peripheral blood derived CD56^dim^CD16^+^ cells revealed more cytotoxicity than CD56^bright^CD16^−^ cells due to higher expression of GzmB and KIR3DL2, which are involved in regulation of cytotoxicity [44]. However, *in vitro* differentiated CD56^+^CD3^−^ NK cells by cytokines in mesenchymal stem cells derived umbilical cord blood indicate reduction of CD16 expression, but have potent cytotoxicity with upregulated expressions of NKG2D, Prf1, NCR44 and GzmB [45]. Similarly, our results showed that CD16 expression was decreased during *in vitro* differentiation of NK cells by IL-15 (data not shown), but expression of genes related to cytotoxicity such as GzmB, Prf1, NCR3 and KIR2DL4 was increased according to increment of CD56 expression (Table 1 and Table S1). Furthermore, PI3K/AKT pathway activated by IL-15 revealed critical pathway to enhance NK cell effector function in KEGG pathway (Table S2) [46]. Therefore, we suggest that *in vitro* differentiated-NK cells may have potent cytotoxicity ability due to induction of cytotoxicity-related genes.

Recently, miRNAs have been widely investigated as master regulators of gene expression during the development and activation of immune cells [15], [16]. In addition, it was reported that changes in specific miRNAs contribute to distinct mRNA expression profiles when rescued tolerant CD8^+^ T cells were preprogrammed to reestablish the tolerant state [34], [35]. Furthermore, we have previously reported that human miR-27a* acts as a negative regulator of NK cell cytotoxicity by silencing Prf1 and GzmB expression. Thus, we focused on miRNAs that regulate marker gene expression during human NK cell differentiation.

As shown in Fig. 2, we found that the expression of miR-150 was strongly increased in mNK cells. Notably, miR-150 was previously identified as a regulator of NK cell development by targeting c-Myb in mice [25]. The mature miR-150 sequence in humans, mice and rats are identical [47]. Thus, we assumed that miR-150 could play an important role in humans and in mice. Additionally, the downregulation of miR-223 in human mNK cells was previously reported to regulate GzmB translation [33]. Therefore, these results suggest that genome-wide miRNA array data could offer important information on the regulation of marker genes related to human NK cell development. However, most miRNAs are novel candidates, the functions of which are unknown in immune cells, including NK cells. Despite the development of various target prediction algorithms, most miRNAs do not necessarily target their predicted target proteins. For this reason, new experimental strategies are required to explore target genes regulated by predicted miRNAs during NK cell differentiation. To identify highly correlated target genes of the four predicted miRNAs in this study, we illustrated a potential network between the four selected miRNAs and their highly correlated target mRNAs using Magia. Following this process, miR-583 showed the largest fold change in mNK cells, and this miRNA was correlated with IL2Rγ, which is a common subunit present in both the IL2 receptor and the IL-15 receptor that stimulates the differentiation and expansion of NK cells. Importantly, IL2Rγ-deficient mice showed a defect in mature T- and B-cell development, as well as a complete lack of NK cell development [36]. Thus, we suggest that miR-583 could act as an essential regulator of IL2Rγ expression during human NK cell development.

Recently, several miRNAs have been closely associated with NK cell development [22]. Bezman et al. examined the expression profile of miRNAs in mouse and human NK cells using microarrays. In mouse NK cells, miR-150 regulated NK cell development by targeting c-Myb [25]. Additionally, miR-155 Tg mice have an increased number of total NK cell and an excess of the CD11b^low^CD27^high^ NK cell subset, which is indicative of a halt in terminal NK cell differentiation; this occurrence proved to be intrinsic to the cell itself, in part via the diminished expression of the inositol phosphatase SHIP-1 [26]. In contrast, miR-155 deficient mice showed a defect in the homeostasis and activation in NK cells [27].

In addition, it has been reported that miR-181 promotes the development of NK cells from CD34^+^ hematopoietic progenitor cells and IFN-γ production in primary human CD56^+^CD3^−^ NK cell, at least in part through the suppression of nemo-like kinase (NLK), an inhibitor of Notch signaling [48]. Thus, miRNAs have been implicated in human NK cell development and activation through their ability to regulate the expression of signature molecules involved in NK cell development. Here, for the first time, we have defined the expression profiles of genome-wide mRNA and miRNAs during human NK cell differentiation. The miR-583 is known to be involved in ZHENG differentiation during chronic Hepatitis B infection through the regulation of the MAPK signaling pathway in liver cells [49], but the effect of this miRNA on immune cell differentiation, including that of NK cells, has not been studied. In this study, we demonstrate that miR-583 regulates the NK cell developmental process by targeting IL2Rγ.

NK cell development requires the acquisition of NK cell-specific receptors and ultimately the acquisition of functional capacities that can act through these receptors, which will be required for NK cells to mediate therapeutic effects in human clinical trials. However, efforts to modulate the cytolytic activities of NK cells against human cancers have not been successful, suggesting that novel targets regulating NK cell development and cytotoxicity must be identified and targeted. The molecular insights into the role of the miRNAs that specifically regulate NK cell differentiation provided by our study suggest that it also may be possible to enhance NK cell–based immunotherapy against human cancers by modulating miRNAs expression during NK cell development.

Collectively, our results provide a comprehensive database of genome-wide mRNA and miRNA expression during human NK cell differentiation, furthering our understanding of basic human NK cell biology for the application of human NK cells in immunotherapy.

## Supporting Information

Table S1 **The molecular signatures involved in human NK cell differentiation.** Genes showing altered more than 2-fold expression in 7d- and 14-d cultured mNK cells compared to 1 d-cultured (24 h) cells. Blue represents down-regulated genes in mNK cells.
(DOCX)

Table S2 **Canonical pathway in NK cell differentiation.** KEGG pathway mapping was performed based on the gene ontology classification in Figure 1c.
(DOCX)

Table S3 **The list of miRNA expression during human NK cell differentiation.** The miRNA microarray was performed using total RNA isolated from 7- or 14- d (mNK) culture cells after differentiation induction. The results are presented from duplicate experiments and deposited in supplemental materials as an Excel file named ‘miRNA profile in human NK differentiation’.
(DOCX)
