# Supplementary material for: Integrated mRNA-MicroRNA Profiling of Human NK Cell Differentiation Identifies MiR-583 as a Negative Regulator of IL2Rγ Expression
Source: PLoS One. 2014 Oct 14;9(10):e108913. doi: 10.1371/journal.pone.0108913 (PMC4196775; doi:10.1371/journal.pone.0108913)
Supplement: Table S1 — The molecular signatures involved in human NK cell differentiation. Genes showing altered more than 2-fold expression in 7d- and 14-d cultured mNK cells compared to 1 d-cultured (24 h) cells. Blue represents down-regulated genes in mNK cells. (DOCX) [file pone.0108913.s001.docx]

**Table S1. The molecular signatures involved in human NK cell differentiation.** Genes showing altered more than 2-fold expression in 7- and 14-d cultured mNK cells compared to 1 d-cultured cells. Blue represents down-regulated genes in mNK cells.

| **Category** | **Gene**  **symbol** | | **Gene Name** | | | **24h vs. 7d** | | **24h vs. mNK** | | **ratio** | **Genbank**  **Acc. No.** | |  |
| --- | --- | --- | --- | --- | --- | --- | --- | --- | --- | --- | --- | --- | --- |
| **Receptor** | SLAMF1 | | signaling lymphocytic activation molecule family member 1 | | | 3.1 | | 65.9 | | 21.4 | NM_003037 | |  |
|  | CXCR3 | | chemokine (C-X-C motif) receptor 3 | | | 2.3 | | 37.2 | | 16.3 | NM_001504 | |  |
|  | KLRD1 | | killer cell lectin-like receptor subfamily D, member 1 | | | 3.0 | | 40.8 | | 13.6 | NM_002262 | |  |
|  | KLRB1 | | killer cell lectin-like receptor subfamily B, member 1 | | | 9.7 | | 84.3 | | 8.7 | NM_002258 | |  |
|  | CD276 | | CD276 molecule | | | 2.0 | | 16.3 | | 8.0 | NM_001024736 | |  |
|  | KLRC1 | | killer cell lectin-like receptor subfamily C, member 1 | | | 9.5 | | 68.5 | | 7.2 | NM_002259 | |  |
|  | IL2RB | | interleukin 2 receptor, beta | | | 10.5 | | 61.3 | | 5.8 | NM_000878 | |  |
|  | CR1 | | complement component (3b/4b) receptor 1 (Knops blood group) | | | 2.6 | | 14.1 | | 5.5 | NM_000651 | |  |
|  | KLRC2 | | killer cell lectin-like receptor subfamily C, member 2 | | | 6.8 | | 35.3 | | 5.2 | NM_002260 | |  |
|  | KLRC4 | | killer cell lectin-like receptor subfamily C, member 4 | | | 3.7 | | 17.9 | | 4.9 | NM_013431 | |  |
|  | NCR1 | | natural cytotoxicity triggering receptor 1 | | | 3.2 | | 14.0 | | 4.4 | BC064806 | |  |
|  | KIR3DL2 | | killer cell immunoglobulin-like receptor, three domains, long cytoplasmic tail, 2 | | | 3.8 | | 16.7 | | 4.4 | NM_006737 | |  |
|  | **NR0B1** | | nuclear receptor subfamily 0, group B, member 1 | | | 0.5 | | 0.1 | | 5.9 | NM_000475 | |  |
| **Cytokines** | XCL1 | | chemokine (C motif) ligand 1 | | | 16.1 | | 126.5 | | 7.9 | NM_002995 | |  |
|  | CCL3 | | chemokine (C-C motif) ligand 3 | | | 2.4 | | 17.7 | | 7.4 | D00044 | |  |
|  | CCL3L3 | | chemokine (C-C motif) ligand 3-like 3 | | | 3.0 | | 21.0 | | 7.0 | NM_001001437 | |  |
|  | CCL13 | | chemokine (C-C motif) ligand 13 | | | 2.5 | | 16.7 | | 6.7 | NM_005408 | |  |
|  | MAL | | mal, T-cell differentiation protein | | | 26.6 | | 74.2 | | 2.8 | NM_002371 | |  |
|  | LIF | | leukemia inhibitory factor (cholinergic differentiation factor) | | | 2.7 | | 13.8 | | 5.0 | NM_002309 | |  |
|  | CXCL9 | | chemokine (C-X-C motif) ligand 9 | | | 33.5 | | 73.2 | | 2.2 | NM_002416 | |  |
| **TF** | TOX | | thymocyte selection-associated high mobility group box | | | 2.0 | | 8.5 | |  | NM_014729 | |  |
|  | GATA3 | | GATA binding protein 3 | | | 2.5 | | 9.4 | | 3.8 | NM_001002295 | |  |
|  | ETS1 | | v-ets erythroblastosis virus E26 oncogene homolog 1 (avian) | | | 2.5 | | 6.6 | | 2.7 | NM_005238 | |  |
|  | EOMES | | eomesodermin homolog (Xenopus laevis) | | | 34.0 | | 72.6 | | 2.1 | NM_005442 | |  |
|  | ID2 | | inhibitor of DNA binding 2, dominant negative helix-loop-helix protein | | | 3.8 | | 7.2 | | 1.9 | NM_002166 | |  |
|  | **PBX1** | | pre-B-cell leukemia homeobox 1 | | | 0.1 | | 0.0 | | 3.7 | NM_002585 | |  |
|  | **HES2** | | hairy and enhancer of split 2 (Drosophila) | | | 0.4 | | 0.2 | | 1.6 | NM_019089 | |  |
|  | **MEF2C** | | myocyte enhancer factor 2C | | | 0.3 | | 0.2 | | 1.3 | NM_002397 | |  |
| **Category** | | **Gene**  **symbol** | | **Gene Name** | **24h vs. 7d** | | **24h vs. mNK** | | **ratio** | | | **Genbank**  **Acc. No.** | |
| **adaptor protein** | | PRF1 | | perforin 1 (pore forming protein) | 5.4 | | 48.6 | | 8.9 | | | NM_005041 | |
|  | | CTSW | | cathepsin W | 8.5 | | 45.4 | | 5.3 | | | NM_001335 | |
|  | | SH2D2A | | SH2 domain protein 2A | 5.5 | | 20.7 | | 3.8 | | | NM_003975 | |
|  | | HLA-DRB5 | | major histocompatibility complex, class II, DR beta 5 | 2.3 | | 8.6 | | 3.8 | | | NM_002125 | |
|  | | GZMB | | granzyme B (granzyme 2, cytotoxic T-lymphocyte-associated serine esterase 1) | 9.7 | | 28.3 | | 2.9 | | | NM_004131 | |
|  | | GZMA | | granzyme A (granzyme 1, cytotoxic T-lymphocyte-associated serine esterase 3) | 39.7 | | 68.5 | | 1.7 | | | NM_006144 | |
|  | | **JRK** | | jerky homolog (mouse) | 0.5 | | 0.0 | | 13.3 | | | NM_003724 | |
|  | | **BCL11A** | | B-cell CLL/lymphoma 11A (zinc finger protein) | 0.4 | | 0.1 | | 3.9 | | | NM_022893 | |
|  | | **ENC1** | | ectodermal-neural cortex (with BTB-like domain) | 0.3 | | 0.1 | | 3.1 | | | NM_003633 | |
| **Kinases** | | PRKCH | | protein kinase C, eta | 3.2 | | 26.5 | | 8.3 | | | NM_006255 | |
|  | | CARD11 | | caspase recruitment domain family, member 11 | 2.1 | | 10.3 | | 4.8 | | | AK097139 | |
|  | | TRIB3 | | tribbles homolog 3 (Drosophila) | 17.1 | | 50.7 | | 3.0 | | | NM_021158 | |
|  | | TIE1 | | tyrosine kinase with immunoglobulin-like and EGF-like domains 1 | 12.6 | | 35.6 | | 2.8 | | | NM_005424 | |
|  | | LCK | | lymphocyte-specific protein tyrosine kinase | 10.3 | | 28.6 | | 2.8 | | | NM_005356 | |
|  | | **CDK14** | | cyclin-dependent kinase 14 | 0.4 | | 0.1 | | 6.0 | | | NM_012395 | |
|  | | **PLK4** | | polo-like kinase 4 (Drosophila) | 0.4 | | 0.1 | | 3.0 | | | NM_014264 | |
| **Phosphatase** | | DUSP5 | | dual specificity phosphatase 5 | 3.4 | | 20.5 | | 6.1 | | | NM_004419 | |
|  | | DUSP6 | | dual specificity phosphatase 6 | 2.2 | | 10.5 | | 4.7 | | | NM_001946 | |
|  | | DUSP2 | | dual specificity phosphatase 2 | 2.9 | | 6.4 | | 2.2 | | | NM_004418 | |
| **proteases** | | GIMAP6 | | GTPase, IMAP family member 6 | 2.1 | | 9.3 | | 4.5 | | | NM_024711 | |
|  | | CAPN12 | | calpain 12 | 5.6 | | 23.9 | | 4.2 | | | NM_144691 | |
|  | | **MSRB3** | | methionine sulfoxide reductase B3 | 0.4 | | 0.1 | | 5.0 | | | NM_001031679 | |
|  | | **HPGDS** | | hematopoietic prostaglandin D synthase | 0.4 | | 0.1 | | 4.6 | | | NM_014485 | |
| **Extracellular**  **matrix** | | FN1 | | fibronectin 1 | 2.5 | | 7.4 | | 3.0 | | | NM_054034 | |
|  | | ICAM1 | | intercellular adhesion molecule 1 | 2.9 | | 4.9 | | 1.7 | | | NM_000201 | |
