# Supplementary material for: Integrated mRNA-MicroRNA Profiling of Human NK Cell Differentiation Identifies MiR-583 as a Negative Regulator of IL2Rγ Expression
Source: PLoS One. 2014 Oct 14;9(10):e108913. doi: 10.1371/journal.pone.0108913 (PMC4196775; doi:10.1371/journal.pone.0108913)
Supplement: Table S2 — Canonical pathway in NK cell differentiation. KEGG pathway mapping was performed based on the gene ontology classification in Figure 1c. (DOCX) [file pone.0108913.s002.docx]

**Table S2. Canonical pathway in NK cell differentiation.**

| KEGG pathways | Annotation | Up-regulated Gene  number/total  (% genes enriched) | Down-regulated Gene number/total  (% genes enriched) |
| --- | --- | --- | --- |
| hsa10018 | PI3K-Akt signaling pathway | 28/64(43.8) | 27/64(42.2) |
| hsa04650 | Natural killer cell mediated cytotoxicity | 19/138(13.8) | 3/138(2.2) |
| hsa04310 | Wnt signaling pathway | 17/151(11.3) | 13/151(8.6) |
| hsa04660 | T cell receptor signaling pathway | 15/108(13.9) | 4/108(3.7) |
| hsa04110 | Cell cycle | 13/124(10.5) | 8/124(6.5) |
| hsa04064 | NFκB signaling pathway | 15/92(16.3) | 7/92(7.6) |
| hsa04810 | Regulation of actin cytoskeleton | 20/213(9.4) | 18/213(8.5) |
| hsa04062 | Chemokine signaling pathway | 24/192(12.5) | 10/192(5.2) |
| hsa04510 | Focal adhesion | 12/204(5.9) | 22/204(10.8) |

Pathway analysis was performed according to the KEGG pathway mapping tool. Up-regulated genes, 1335; down-regulated genes, 1585.
