# Supplementary material for: Integrated mRNA-MicroRNA Profiling of Human NK Cell Differentiation Identifies MiR-583 as a Negative Regulator of IL2Rγ Expression
Source: PLoS One. 2014 Oct 14;9(10):e108913. doi: 10.1371/journal.pone.0108913 (PMC4196775; doi:10.1371/journal.pone.0108913)
Supplement: Table S3 — The list of miRNA expression during human NK cell differentiation. The miRNA microarray was performed using total RNA isolated from 7- or 14- d (mNK) culture cells after differentiation induction. The results are presented from duplicate experiments and deposited in supplemental materials as an Excel file named ‘miRNA profile in human NK differentiation’. (DOCX) [file pone.0108913.s003.docx]

**Table S3.** The list of miRNA expression during human NK cell differentiation. The miRNA microarray was performed using total RNA isolated from 7- or 14- d (mNK) culture cells after differentiation induction. The results are presented from duplicate experiments (1 and 2).
